# Supplementary material for: Crosstalks between mTORC1 and mTORC2 variagate cytokine signaling to control NK maturation and effector function
Source: Nat Commun. 2018 Nov 19;9:4874. doi: 10.1038/s41467-018-07277-9 (PMC6242843; doi:10.1038/s41467-018-07277-9)
Supplement: Supplementary file 1 — Supplementary Information [file 41467_2018_7277_MOESM1_ESM.docx]

**Crosstalks between mTORC1 and mTORC2 variagate cytokine signaling to control NK maturation and effector function**

Wang et al.

**SUPPLEMENTARY MATERIALS**

**
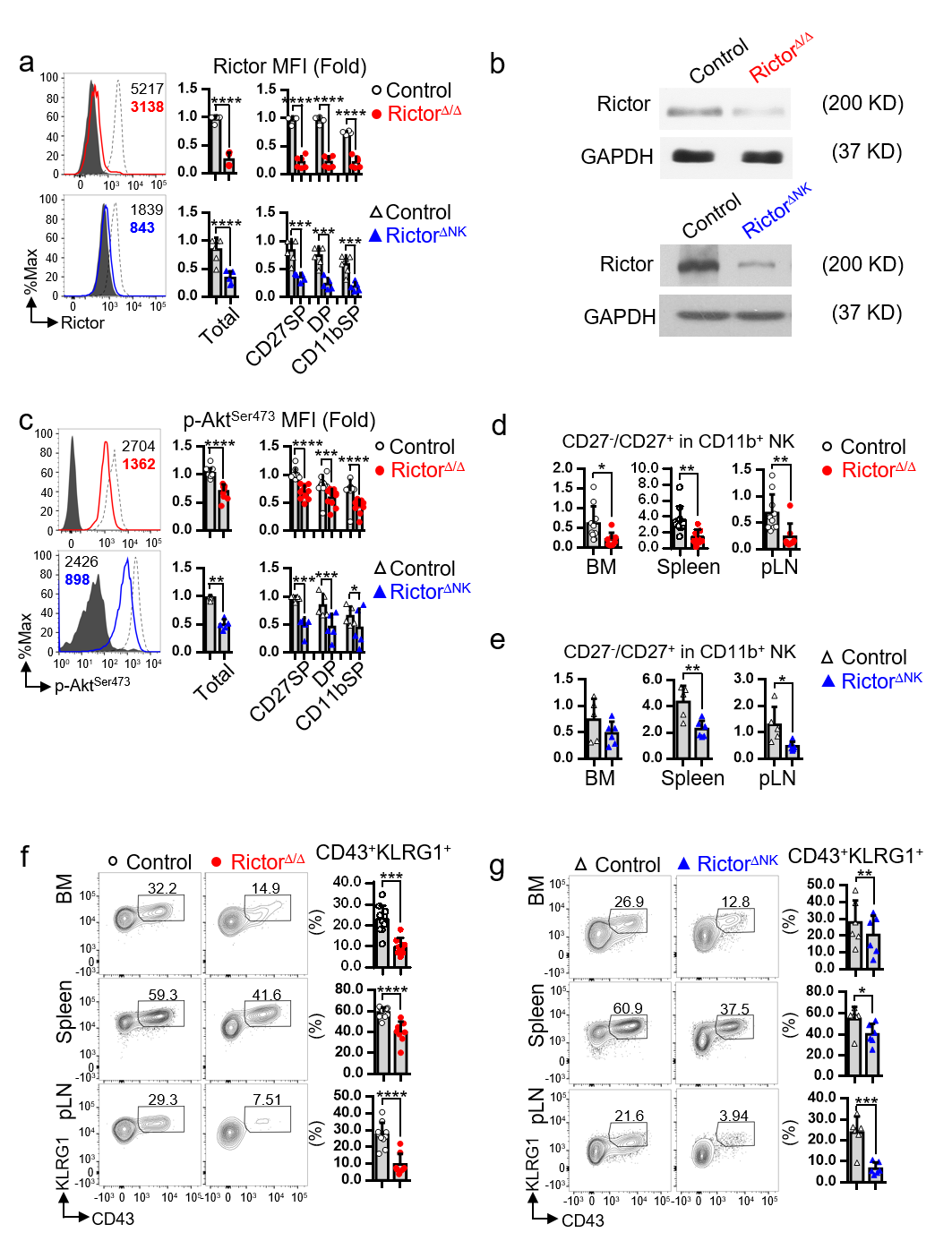
**

**Supplementary Figure 1** Rictor deficiency impairs NK cell maturation. (**a, c**) Intracellular flow cytometric analysis and cumulative results depicting Rictor expression (**a**) and phosphorylation (p-) of Akt at Ser473 (p-Akt^Ser473^) (**c**) in total splenic NK cells (CD3^―^CD19^―^NK1.1^+^) and subpopulations thereof from control (*Rictor*^fl/fl^/*Vav1*-Cre^―^) *versus* Rictor^∆/∆^ (*Rictor*^fl/fl^/*Vav1*-Cre^+^) (top) or control (*Rictor*^fl/+^/*Ncr1*-Cre^+^) *versus* Rictor^∆NK^ (*Rictor*^fl/fl^/*Ncr1*-Cre^+^) (bottom) mice. (**b**) Immunoblotting for Rictor expression in purified splenic NK cells from control *versus* Rictor^∆/∆^ (top) or control *versus* Rictor^∆NK^ (bottom) mice, with GAPDH serving as an internal control. (**d, e**) The calculated ratio of CD27^―^ *versus* CD27^+^ cells among CD11b^+^ NK cells in the BM, spleen and peripheral lymph nodes (pLNs) from control *versus* Rictor^∆/∆^ (**d**) or control *versus* Rictor^∆NK^ (**e**) mice, analyzed by flow cytometry. (**f, g**) Flow cytometric analysis and cumulative frequencies depicting the CD43^+^KLRG1^+^ subset of NK cells (CD3^―^CD19^―^NK1.1^+^NKp46^+^) in the BM, spleen and pLNs from control *versus* Rictor^∆/∆^ (**f**) or control *versus* Rictor^∆NK^ mice (**g**). In the histograms, the dashed line indicates the control group, and the solid line indicates the gene knockout group. The MFI is relative to total NK cells or the CD27SP subset of control littermates. The subpopulations of NK cells are distinguished by CD27 and CD11b, and CD27SP, DP, and CD11bSP represent CD27^+^CD11b^―^, CD27^+^CD11b^+^, and CD27^―^CD11b^+^ NK cell subsets, respectively. For the bar graphs, each dot represents one mouse in experiments that were replicated 3 (**a**, **c** **bottom**), 4 (**e**, **g**), 5 (**d**, **f**), 6 (**c top**) times. Error bars represent SD; ∗p < 0.05, ∗∗p < 0.01, ∗∗∗p < 0.001, and ∗∗∗∗p < 0.0001; unpaired two-tailed Student’s t-test with Welsh’s correction (**a**, **c-g**).

**
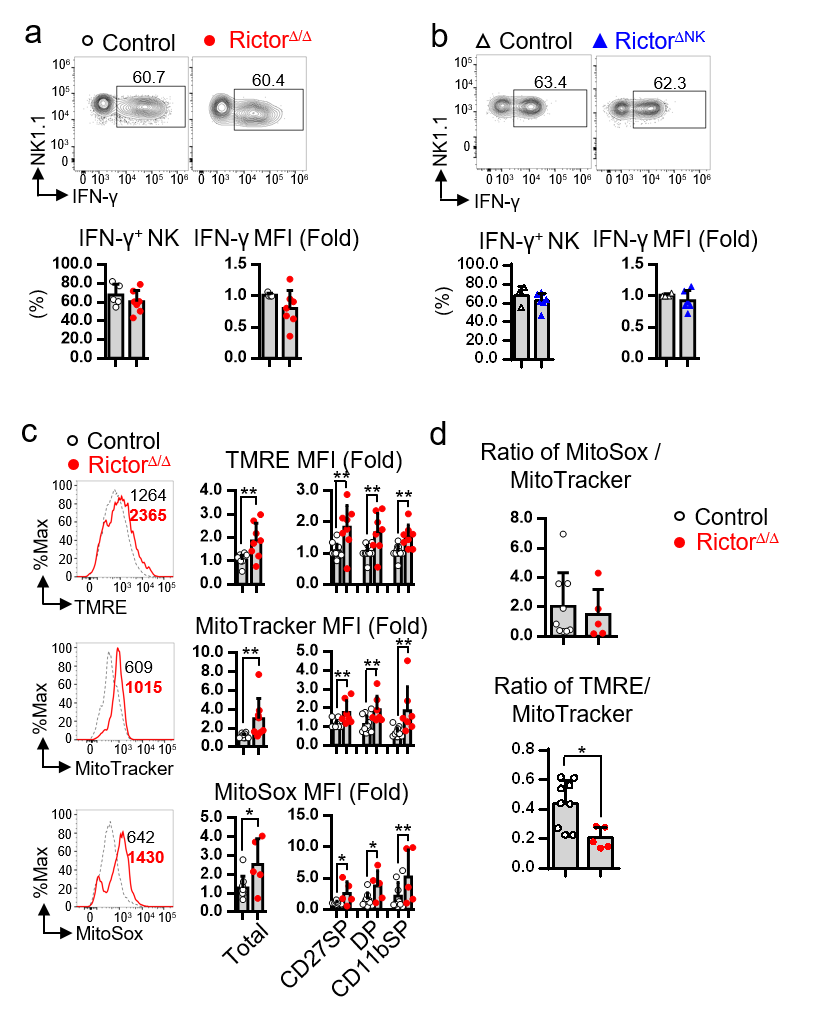
**

**Supplementary Figure 2** mTORC2 deficiency promotes NK cell effector functions. (**a, b**) Intracellular flow cytometric analysis and cumulative frequencies of IFN-γ^+^ splenic NK cells (CD3^―^CD19^―^ NK1.1^+^NKp46^+^) (left) and the MFI of IFN-γ in IFN-γ^+^ NK cells (right) from control *versus* Rictor^∆/∆^ (**a**) or control *versus* Rictor^∆NK^ (**b**) mice, following stimulation with PMA and ionomycin in the presence of GolgiPlug for 6 hours. (**c**) Flow cytometric analysis depicting TMRE, MitoTracker, and MitoSox staining of splenic NK cells (CD3^―^CD19^―^ NK1.1^+^ NKp46^+^) and the subpopulations thereof. (**d**) The calculated ratio of the MFI of TMRE / MFI of MitoTracker and the MFI of MitoSox / MFI of MitoTracker in splenic NK cells for (**c**). The MFI is calculated relative to total NK cells or to the CD27SP subset from control littermates. Subpopulations of NK cells are distinguished by CD27 and CD11b expression, and CD27SP, DP, and CD11bSP represent CD27^+^CD11b^―^, CD27^+^CD11b^+^, and CD27^―^CD11b^+^ NK cell subsets, respectively. For the bar graphs, each dot represents one mouse in experiments that were replicated 3 (**b**), 4 (**a**), 5 (**c**, **d**) times. Error bars represent SD; ∗p < 0.05, ∗∗p < 0.01, ∗∗∗p < 0.001, and ∗∗∗∗p < 0.0001; unpaired two-tailed Student’s t-test with Welsh’s correction (**a**-**d**).


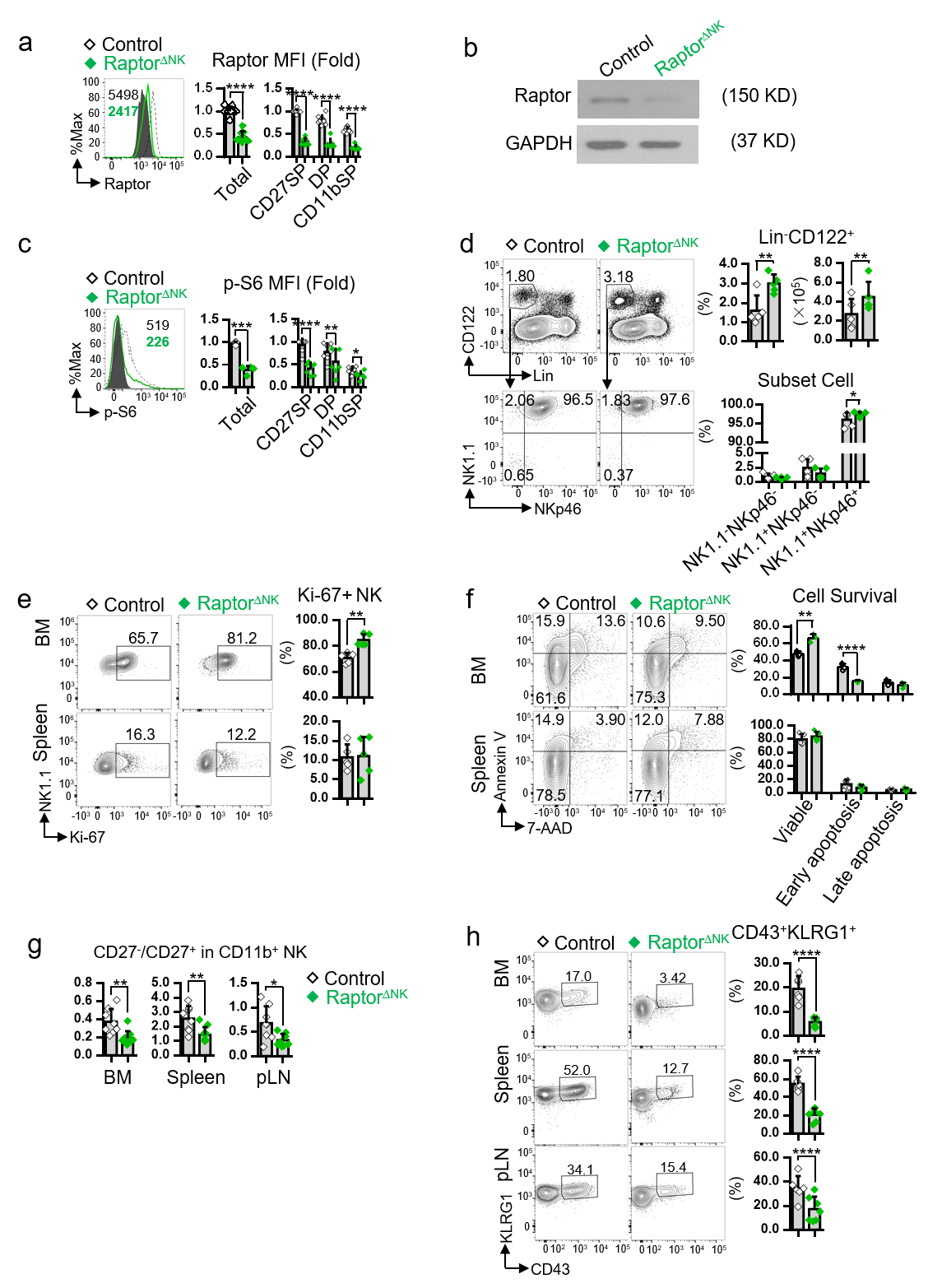


**Supplementary Figure 3** mTORC1 regulates NK cell homeostasis in the BM as well as NK cell maturation and antitumor activity. (**a, c**) Intracellular flow cytometric analysis and cumulative results depicting Raptor expression (**a**) and phosphorylation (p-) of S6 at ser235/236 (p-S6^ser235/236^) (**c**) in total splenic NK cells (CD3^―^CD19^―^NK1.1^+^) and subpopulations thereof from control (*Rptor*^fl/+^/*Ncr1*-Cre^+^) *versus* Raptor^∆NK^ (*Rptor*^fl/fl^/*Ncr1*-Cre^+^) mice. (**b**) Immunoblotting for Raptor expression in purified splenic NK cells from control *versus* Raptor^∆NK^ mice, with GAPDH serving as an internal control. (**d**) Flow cytometric analysis and enumeration of Lin^−^CD122^+^ (Lin: CD3, CD19, Ter119, Gr1) BM cells (top) and the percentage of subsets expressing NK1.1 and/or NKp46 belonging to Lin^―^CD122^+^ BM cells (bottom). (**e**) Intracellular flow cytometric analysis and cumulative results depicting the percentage of Ki-67^+^ cells among total NK cells (CD3^―^CD19^―^NK1.1^+^NKp46^+^) from the BM and spleen of control *versus* Raptor^∆NK^ mice. (**f**) Flow cytometric analysis and cumulative apoptosis of BM and splenic NK cells (CD3^―^CD19^―^NK1.1^+^NKp46^+^) from control *versus* Raptor^∆NK^ mice. Viable cells are represented by the 7-AAD^―^Annexin V^―^ subset, early apoptotic cells are represented by the 7-AAD^―^ Annexin V^+^ subset, late apoptotic cells are represented by the 7-AAD^+^Annexin V^+^ subset. (**g**) The calculated ratio of CD27^―^ *versus* CD27^+^ cells among CD11b^+^ NK cells in the BM, spleen and peripheral lymph nodes (pLNs) from control *versus* Raptor^∆NK^ mice analyzed by flow cytometry. (**h**) Flow cytometric analysis and cumulative frequencies of the CD43^+^KLRG1^+^ subset of NK cells (CD3^―^CD19^―^NK1.1^+^NKp46^+^) in the BM, spleen and pLNs from control *versus* Raptor^∆NK^ mice. The MFI was calculated relative to total NK cells or the CD27SP subset from control littermates. Subpopulations of NK cells are distinguished by CD27 and CD11b expression, and CD27SP, DP, and CD11bSP represent CD27^+^CD11b^―^, CD27^+^CD11b^+^, and CD27^―^CD11b^+^ NK cell subsets, respectively. For the bar graphs, each dot represents one mouse in experiments that were replicated 3 (**c-f**, **h**), 4 (**g**), 7 (**a**) times. Error bars represent SD; ∗p < 0.05, ∗∗p < 0.01, ∗∗∗p < 0.001, and ∗∗∗∗p < 0.0001; unpaired two-tailed Student’s t-test with Welsh’s correction (**a, c-h**).

**
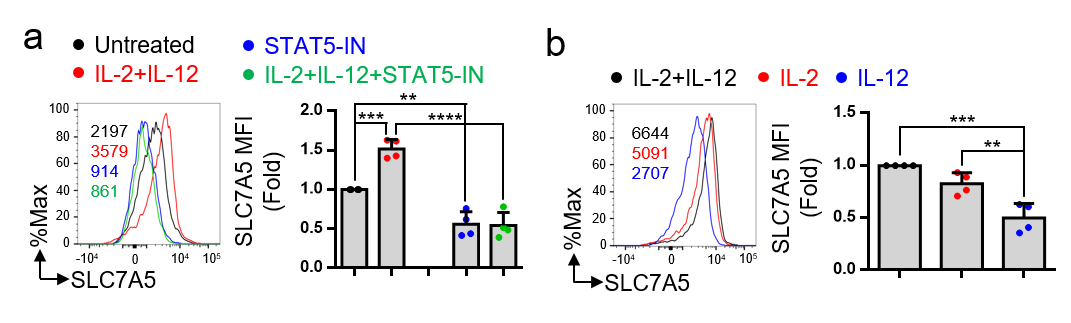
**

**Supplementary Figure 4** Validation of SLC7A5 antibodies for flow cytometric analysis. (**a**) SLC7A5 protein levels were analyzed by flow cytometry after WT NK cells being left untreated or being stimulated with IL-2 (20 ng/ml) + IL-12 (10 ng/ml) and with or without STAT5-IN for 18 h. (**b**) WT NK cells were cocultured with IL-2 (20 ng/ml) + IL-12 (10 ng/ml) for 20 h and then switched into media containing only IL-2 (20 ng/ml) or IL-12 (10 ng/ml) for another 8 h. SLC7A5 protein levels were then analyzed by flow cytometry after treatment. For the bar graphs, each dot represents one individual mouse in experiments (**a, b**). Error bars represent SD; ∗∗p < 0.01, ∗∗∗p < 0.001, and ∗∗∗∗p < 0.0001; one-way ANOVA (**a**, **b**).


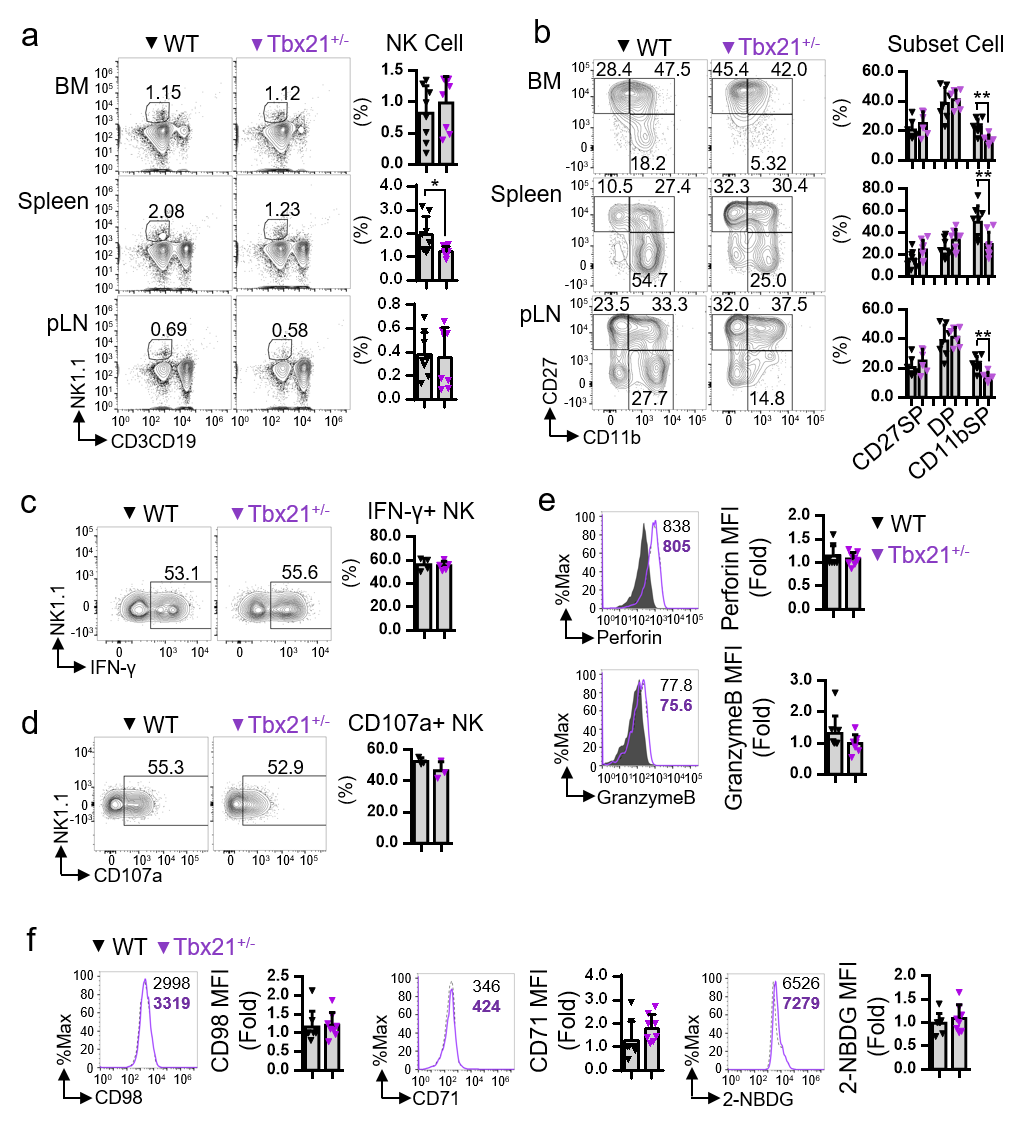


**Supplementary Figure 5** Single allele expression of Tbx21 was insufficient to maintain NK cell development but was sufficient for acquisition of NK cell effector functions in Tbx21^+/―^ mice. (**a**) Flow cytometric analysis and cumulative results depict the ratio of NK cells (CD3^―^CD19^―^NK1.1^+^) relative to total lymphocytes in the BM, spleen and peripheral lymph nodes (pLNs). (**b**) Flow cytometric analysis and cumulative frequencies of subpopulations of NK cells (CD3^―^CD19^―^NK1.1^+^NKp46^+^) in the BM, spleen and pLNs. (**c**) Intracellular flow cytometric analysis and cumulative frequencies of IFN-γ^+^ splenic NK cells (CD3^―^CD19^―^NK1.1^+^NKp46^+^) following stimulation with PMA and ionomycin in the presence of GolgiPlug for 6 hours. (**d**) Flow cytometric analysis and cumulative frequencies of CD107a^+^ splenic NK cells (CD3^―^CD19^―^NK1.1^+^NKp46^+^) after coculture with Yac-1 target cells in the presence of GolgiStop for 6 hours. (**e, f**) Flow cytometric analysis and cumulative results depicting the fold change in perforin and granzyme B expression (**e**), in CD98 and CD71 expression, and in 2-NBDG uptake (**f**) by splenic NK cells (CD3^―^CD19^―^ NK1.1^+^ NKp46^+^). For the histograms, the dashed line indicates the control group, and the solid line indicates the gene knockout group. The MFI was calculated relative to total NK cells. Subpopulations of NK cells are distinguished by CD27 and CD11b expression, and CD27SP, DP, and CD11bSP represent CD27^+^CD11b^―^, CD27^+^CD11b^+^, and CD27^―^CD11b^+^ NK cell subsets, respectively. For the bar graphs, each dot represents one mouse in experiments that were replicated 3 (**b**-**d**), 4 (**a**, **e**, **f**) times; Error bars represent SD; ∗p < 0.05, and ∗∗p < 0.01; unpaired two-tailed Student’s t-test with Welsh’s correction (**a**-**f**).


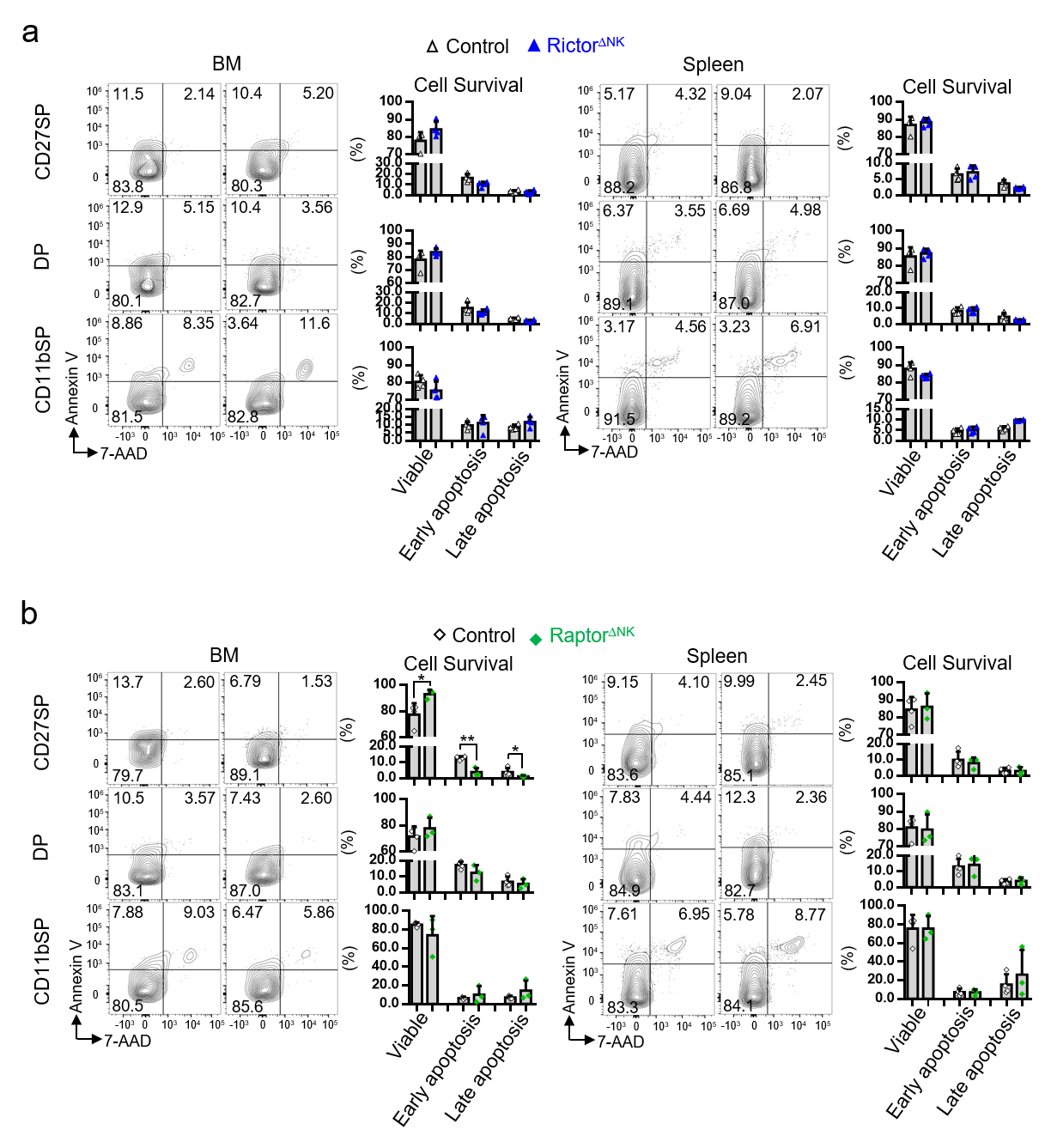


**Supplementary Figure 6** The viability of Rictor or Raptor deficient NK cell subsets. (**a, b**) Flow cytometric analysis of NK cell subsets viability from BM and spleen in control *versus* Rictor^∆NK^ (**a**) or control *versus* Raptor^∆NK^ (**b**) mice. Viable cells are represented by the 7-AAD^―^Annexin V^―^ subset; early apoptotic cells are represented by the 7-AAD^―^ Annexin V^+^ subset; while late apoptotic cells are represented by the 7-AAD^+^Annexin V^+^ subset. Subpopulations of NK cells are distinguished by CD27 and CD11b expression, and CD27SP, DP, and CD11bSP represent CD27^+^CD11b^―^, CD27^+^CD11b^+^, and CD27^―^CD11b^+^ NK cell subsets, respectively. For the bar graphs, each dot represents one mouse in experiments that were replicated 3 times (**a**, **b**); Error bars represent SD; ∗p < 0.05, and ∗∗p < 0.01; unpaired two-tailed Student’s t-test with Welsh’s correction (**a**, **b**).


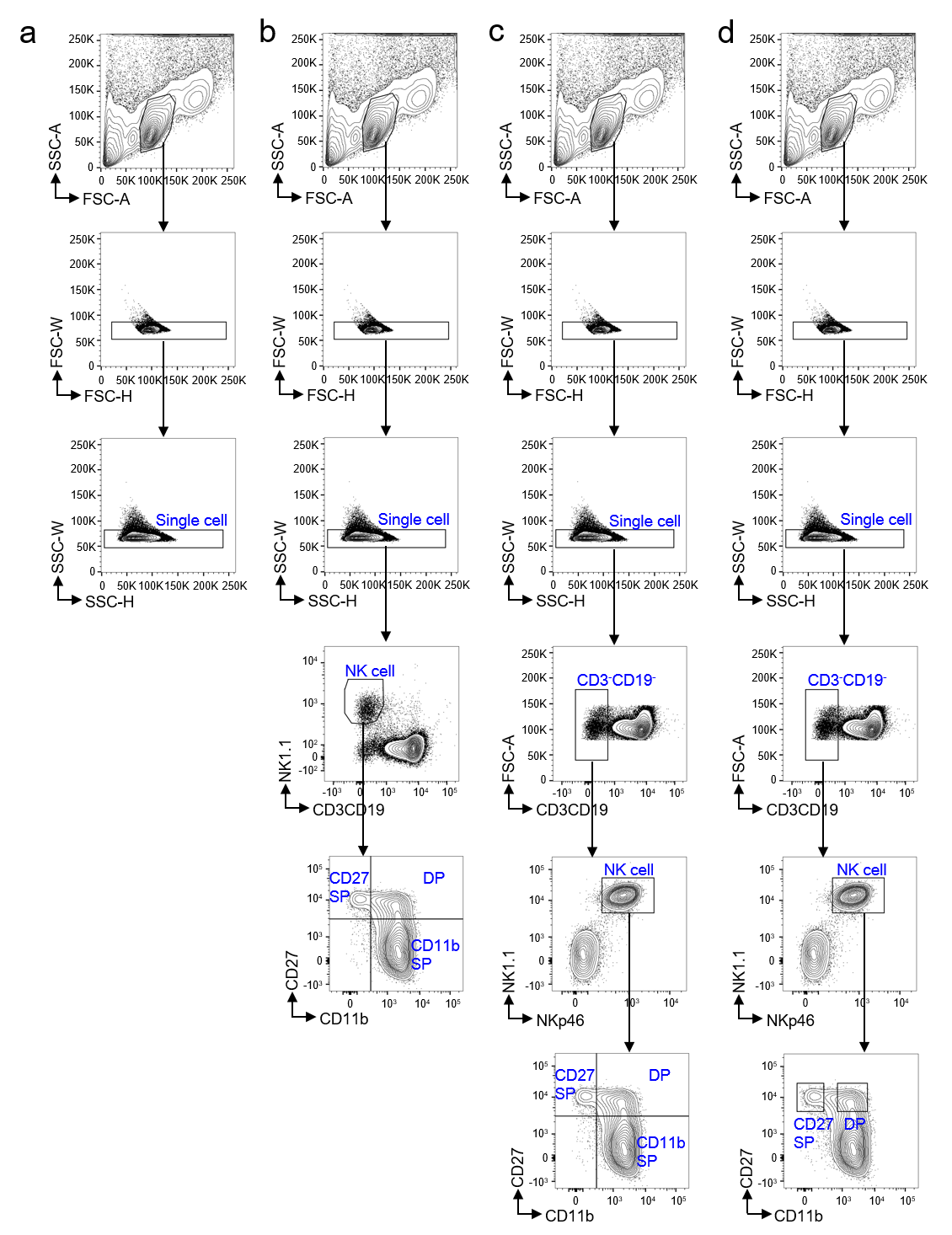


**Supplementary Figure 7** The sequential gating or sorting strategies for flow cytometry. (**a**) The gating strategies for Fig. 1a-d, Fig. 3a, Fig. 5b, Supplementary Fig. 3d, Supplementary Fig. 5a. (**b**) The gating strategies for Fig. 4b, c, e, f, g, Fig. 5a, Fig. 6d, Fig. 7a, b, d, e, j, Supplementary Fig. 1c, Supplementary Fig. 3c. (**c**) The gating strategies for Fig. 1e, f, Fig. 2a, c, d, f-h, Fig. 3b-d, f, g, Fig. 4d, Fig. 5c-g, Fig. 6a-c, e-g, Fig. 7c, f-i, Supplementary Fig. 1a, f, g, Supplementary Fig. 2a-c, Supplementary Fig. 3a, e-h, Supplementary Fig. 4a, b, Supplementary Fig. 5b-f, Supplementary Fig. 6a, b. (**d**) The sorting strategies for Fig. 2b.


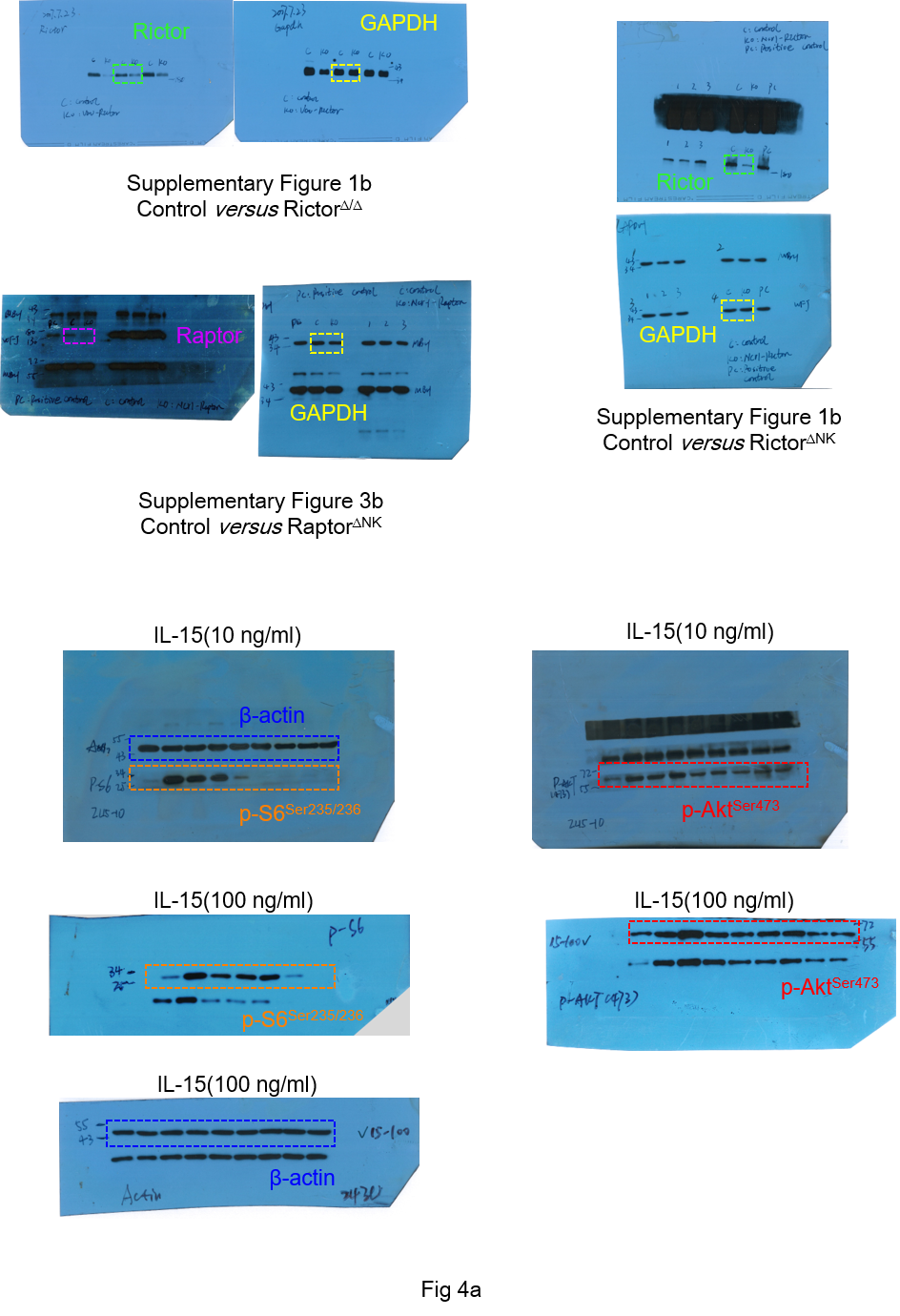


**Supplementary Figure 8** Uncropped version of Western blots in Supplementary Fig 1b, Supplementary Fig 3b and Fig 4a.
